# Lower limb axonal mononeuropathies as sequelae of COVID-19: a case report and review of literature

**DOI:** 10.1186/s41983-022-00458-w

**Published:** 2022-02-14

**Authors:** Saad Bin Zafar Mahmood, Muhammad Zain Mushtaq, Dureshahwar Kanwar, Syed Ahsan Ali

**Affiliations:** Department of Medicine, Aga Khan University Hospital, Stadium Road, Karachi, Pakistan

**Keywords:** COVID-19, Mononeuropathies, Peripheral neuropathy, Complication

## Abstract

**Background:**

Neurological symptoms and complications of Coronavirus disease 2019 (COVID-19) were seldom discussed in the literature initially. Neurological symptoms such as headache, dizziness, anosmia, hypogeusia, and neuralgia are, however, now being reported commonly. Mononeuropathies are rare complications of COVID-19, with most cases associated with prolonged intensive care stay.

**Case presentation:**

A 61-year-old gentleman with prior history of well-controlled diabetes and hypertension was recently treated for COVID-19 pneumonia with supplemental oxygen and positive pressure ventilation. He now presented with left-sided foot weakness two weeks after recovering from the viral illness. On examination he had normal bulk and tone and a power of 4/5 in proximal and distal muscles of bilateral lower limbs except for ankle dorsiflexion on the left which was 2/5. He also had absent ankle and knee reflexes bilaterally with bilateral flexor plantar reflexes. Since the patient had no back pain and the sensory system was normal, the lesion was localized to the peripheral nerves and a Nerve Conduction Studies and Electromyography (NCS/EMG) was done. NCS/EMG showed findings suggestive of axonal mononeuropathies. Relevant workup done to identify the cause of mononeuropathy was negative including infectious and autoimmune workup. Since diabetes was well-controlled and he had no intensive care stay his findings were presumed to be associated with resolving COVID-19 infection. The patient underwent aggressive daily physical therapy and has started to show improvement in symptoms.

**Conclusions:**

Complications such as mononeuropathies should be kept in mind in patients recovering from COVID-19 infection, since timely diagnosis can improve clinical outcomes in patients.

## Introduction

The clinical course of Coronavirus disease 2019 (COVID-19) ranges from asymptomatic infection to severe acute respiratory distress with multi-organ involvement and death. Majority of the literature focuses mainly on pulmonary complications of the disease [1]. Neurological symptoms such as headache, dizziness, anosmia, hypogeusia, and neuralgia are, however, now being reported commonly [2]. Most of the complications associated with the peripheral nervous system have revolved around Guillain–Barre syndrome (GBS) and its variants involving cranial nerves [3]. Manifestation of peripheral motor neuropathy is rare, and most cases are associated with prolonged intensive care unit stay [4]. Here we present a rare presentation of bilateral lower limb axonal mononeuropathies 2 weeks after recovery from COVID-19 pneumonia. Although, peripheral neuropathy is a rare manifestation of COVID-19, this complication should be present in the mind of the treating physician dealing with this disease. This will lead to timely diagnosis of such rare complications and better outcomes of such patients.

## Case presentation

A 61-year-old male with prior history of well controlled diabetes on oral hypoglycemic drugs and hypertension presented with complaints of fever, cough, and shortness of breath for last 10 days with no other systemic complaints. On examination he was tachypneic, tachycardic, febrile and had an oxygen saturation of 90% at an FiO_2_ of 35%. He underwent a nasopharyngeal polymerase chain reaction (PCR) test for SARS-CoV-2 which was positive. He required non-invasive ventilation (NIV) and was categorized as critical COVID-19 pneumonia. He received high dose dexamethasone and piperacillin/tazobactam. A single dose of tocilizumab was given in view of raised inflammatory markers and cytokine release syndrome (CRS). The patient also underwent sessions of awake proning as much as tolerable. He was tapered off from NIV within 2 days and was kept on supplemental oxygen. His steroids were tapered on discharge. Hospital stay was complicated due to development of spontaneous pneumomediastinum and surgical emphysema. Apart from pneumomediastinum and surgical emphysema he made an unremarkable recovery and was discharged on 10th day of hospitalization.

Two weeks later, he presented to the outpatient clinic for follow-up and still complained of mild shortness of breath on exertion. Moreover, he complained of new onset left sided foot weakness. On examination he had normal bulk and tone and a power of 4/5 in proximal and distal muscles of bilateral lower limbs except for ankle dorsiflexion on the left which was 2/5. He also had absent ankle and knee reflexes bilaterally with bilateral flexor plantar reflexes. The rest of the neurological examination was normal.

Our differential diagnoses for the left foot weakness initially were either an acute mononeuropathy likely sciatic/peroneal or an acute L5 radiculopathy. The presence of normal tone and non-progressive localized weakness was against the diagnosis of GBS. Since patient had no back pain and his symptoms were localized to peripheral nerves sparing the sensory system no imaging of spinal cord was done. In view of the clinical exam, we localized the lesion to the peripheral nerves and a Nerve Conduction Studies and Electromyography (NCS/EMG) was requested (Nicolet® VikingQuest® EMG/NCS/EP System, USA). NCS revealed an absent left superficial peroneal response on sensory nerve conduction studies with a normal corresponding right response and normal sural nerves. On motor NCS, the nerve conduction velocities were slow in bilateral peroneal nerves recorded from extensor digitorum brevis muscles bilaterally with absent F waves and normal latencies and compound muscle action potentials (CMAP’s). NCS from bilateral peroneal and tibialis anterior was within normal limits. Bilateral tibial nerves recorded from abductor hallucis showed decreased CMAP’s with normal latencies but slow conduction velocities and absent F waves. The H reflex was absent bilaterally.

On needle EMG, active denervation including fibrillations and positive sharp waves were seen only in the peroneal innervated muscles bilaterally with reduced recruitment including bilateral tibialis anterior, extensor digitorum brevis, extensor hallucis longus and peroneus longus muscles. The upper extremities nerve conduction studies and needle EMG were within normal limits. The above-mentioned findings of NCS taken together were suggestive of acute bilateral tibial mononeuropathies at non compressible sites that was moderate in degree electrically along with acute bilateral common peroneal mononeuropathies of axonal type localized proximal to the branch innervating the peroneus longus muscles bilaterally and severe in degree bilaterally.

The algorithm described by Lehmann et al. was followed for identification of the cause behind the axonal mononeuropathies in our case and relevant workup was sent, as summarized in Table 1 [5]. Neuropathies due to diabetes and critical illnesses were important differentials. However, since our patient had a well-controlled diabetes of short duration and had asymmetrical findings and normal sural nerves on NCS, it was less likely that his symptoms were related to diabetes. Similarly, our patient had no intensive care stay and was on NIV for just 48 h with minimal inspiratory pressure. Therefore, diagnosis of critical illness neuropathy was also less likely. Patient also did not have any significant drug history apart from oral hypoglycemics and angiotensin–receptor blocker and lacked exposure to any heavy metal and occupational risk factors. Antinuclear antibodies were negative making autoimmune causes less likely, while diseases such as amyloidosis are more likely to result in a multisystem disease unlike our case, hence testing for these were not done. Infectious causes such as human immunodeficiency virus and hepatitis C were ruled out. Metabolic abnormalities such as thyroid disorders and vitamin deficiencies were also checked. Patients had no history of malignancy, making paraneoplastic causes of neuropathy least likely.

**Table 1:** Investigations done to identify the etiology of mononeuropathy

Tests	Results	Normal range
Hemoglobin A1c (%)	6.2	≤ 6.5
Thyroid stimulating hormone (mIU/L)	3.3	0.5–8.9
Serum B12 (ng/L)	268	> 250
Serum folate (ng/ml)	4.9	2.5–13.5
Human immunodeficiency virus antibody	Negative	
Hepatitis C antibody	Negative	
Antinuclear antibodies	Negative	

Unfortunately, due to unavailability of tests at our center, we were not able to perform further testing for immune mediated causes such as anti-ganglioside antibody, anti-neurofascin and contactin-1 antibody, and Caspr1/2 antibodies. We were also unable to perform nerve ultrasound to confirm diagnosis of immune-mediated peripheral neuropathies.

Keeping in view of all these findings these bilateral mononeuropathies confined to the lower extremities were most likely associated with resolving COVID-19 infection. He was later referred to a rehabilitation center for his lower limb axonal mononeuropathies and symptomatic treatment along with aggressive daily physical therapy was advised. He made complete recovery from COVID-19 pneumonia after 3 months and now has no residual respiratory symptoms. After extensive physical therapy he has made a remarkable recovery and his left foot weakness is better, while power in ankle dorsiflexion is now 4/5. His last follow-up was 1 month back, 6 months onwards from the COVID-19 infection.

## Discussion

Neurological complications are not as rare as previously believed and both central and peripheral nervous system are being affected by COVID-19 either directly or indirectly [6]. Complications involving the peripheral nervous system are mostly olfactory and gustatory in nature [7]. Few reports have surfaced mentioning GBS and other neuromuscular disorders [6]. Another case series of 214 patients showed that symptoms of peripheral nervous system had an occurrence of 8.9% [8]. However, peripheral neuropathy has a rare association with COVID-19 with an estimated prevalence of less than 1% in a recent study [9]. Pakistan has reported an approximate 18.9% cases with neurological complications; however, no case of neuropathy has been reported from here [10].

Here we have discussed a case of a middle-aged man who developed axonal mononeuropathies as a sequela of COVID-19, an exceedingly rare complication seen in this disease. Majority of the case series and studies have shown that symptoms of peripheral neuropathy appeared along with the respiratory symptoms [7, 11]. One study has reported that the neurological symptoms preceded the respiratory symptoms [12]. However, some case reports and studies showed that neuropathy started after the respiratory symptoms started resolving as in our case [4, 13, 14]. Nonetheless, most of the cases with these symptoms of peripheral neuropathy had prolonged intensive care stay which was not present in our case [4, 7].

The presentation of mononeuropathies in COVID-19 patients has a vast spectrum. Patients have presented with quadriparesis, paraplegia, symmetrical and asymmetrical weaknesses [15], neuropathic pain [16], fatigue and myalgia [14], and numbness [13]. Moreover, Literature review shows that peripheral neuropathy in COVID-19 patients can be of both axonal and demyelinating type. The largest case series of 14 COVID-19 patients with mononeuropathies had characteristic axonal type of pathology [4], while another case series of three patients reported demyelinating lesions [14]. Three studies had no NCS/EMG done due to improvement in symptoms and COVID-19 isolation policies [11–13]. Our patient presented with asymmetrical weakness localized to lower limbs characterized as axonal mononeuropathies and also had no associated pain or other sensory symptoms, nor had any symptoms of dysautonomia. Even though, our patient had no sensory system, NCS revealed an absent left superficial peroneal response on sensory nerve conduction studies. It is very common that the clinical exam does not always match the nerve conduction studies. To give an example, in Guillain Barre syndrome the patient has no abnormalities on the clinical exam; however, the sensory NCS are found to be abnormal frequently [17]. Similarly, in patients with lumbar radiculopathy, findings of clinical exam, NCS and radiological investigations do not always match [18].

The mechanism behind peripheral neuropathy remains unclear. There are two school of thoughts concerning the mechanism behind peripheral neuropathy in COVID-19 patients. The axonal or demyelinating lesion may be depending upon the mechanism behind the injury. Immune-mediated mechanism is one of the hypothesized theories to be behind peripheral neuropathies in cases of COVID-19. It has been seen that the COVID-19 virus shares two hexapeptides with human shock proteins 90 and 60. Both proteins have immunogenic potentials, leading to slow damage of the axons and causing axonal mononeuropathies as in our case and three other studies [3, 13, 15]. The other theory has been hypothesized by Guadarrama-Ortiz et al., stating that vascular, thrombotic, ischemic, and direct nerve effects driven by SARS-CoV-2 can also lead to neuropathy in COVID-19 patients [14, 16]. The same study also states that early neurological signs may be indicative of a direct injury on the peripheral nerves from the virus. In our case, however, this may not be the mechanism behind as the symptoms of mononeuropathies appeared after the resolution of respiratory symptoms.

It is essential to ascertain the cause of mononeuropathies before associating it with COVID-19 infection. Diabetes and critical illness neuropathy remain important causes. Literature shows that distal symmetric sensorimotor polyneuropathy is the most common form of neuropathy seen in diabetes because of “dying back” axonal degeneration [19]. Another characteristic finding seen in diabetic neuropathy is the involvement of sural and tibial nerves. Furthermore, the incidence of neuropathic symptoms is more likely to be increased with uncontrolled and long duration of diabetes [20, 21]. Critical illness neuropathy is a diffuse process; hence, findings are expected to be seen symmetrically. As seen in diabetes, sural nerve involvement is also characteristically seen in critical illness neuropathy [22]. High inspiratory pressures, increased ventilator days and longer intensive care stay are also important characteristics of critical illness neuropathy [23]. However, the characteristics seen in these two causes are quite different than our case as diabetes was well controlled and short of duration and the patient had no stay in intensive care, while NIV was only applied for 48 h. Furthermore, findings on nerve conduction studies were of asymmetrical nature and spared the sural nerves.

Treatment in our case was extensive physical therapy and rehabilitation. Six months later he showed remarkable improvement. This is in line with majority of the cases reported in literature which emphasize upon the importance of effective rehabilitation in these patients. Plasma exchange has also helped in rapid resolution of neurological as well as respiratory symptoms [11].

## Conclusions

Neurological complications of COVID-19 should be kept in mind even after resolution of respiratory symptoms. Peripheral neuropathies especially axonal mononeuropathies are rare but important complications of COVID-19 infection. Timely diagnosis of these presentations is important for effective management and can help in improving clinical outcomes of patients.

## Data Availability

Not applicable.

## Abbreviations

CMAP’s: Compound muscle action potentials
COVID-19: Coronavirus disease 2019
CRS: Cytokine release syndrome
GBS: Guillain–Barre syndrome
NCS/EMG: Nerve conduction studies and electromyography
NIV: Non-invasive ventilation
PCR: Polymerase chain reaction
